# Nanocomposite of electrochemically reduced graphene oxide and gold nanourchins for electrochemical DNA detection

**DOI:** 10.1049/nbt2.12086

**Published:** 2022-04-20

**Authors:** Mostafa Azimzadeh, Zahra Aghili, Behrooz Jannat, Saeid Jafari, Saeed Rafizadeh Tafti, Navid Nasirizadeh

**Affiliations:** ^1^ Halal Research Center of IRI, MOH Tehran Iran; ^2^ Medical Nanotechnology & Tissue Engineering Research Center Yazd Reproductive Sciences Institute Shahid Sadoughi University of Medical Sciences Yazd Iran; ^3^ Stem Cell Biology Research Center Yazd Reproductive Sciences Institute Shahid Sadoughi University of Medical Sciences Yazd Iran; ^4^ Department of Advanced Medical Sciences and Technologies School of Paramedicine Shahid Sadoughi University of Medical Sciences Yazd Iran; ^5^ Food & Drug Control Reference Laboratories Center, FDA, MOH Tehran Iran; ^6^ Department of Textile and Polymer Engineering, Yazd Branch Islamic Azad University Yazd Iran

**Keywords:** biosensors, DNA, electrochemical sensors, nanocomposites

## Abstract

A nanocomposite of graphene oxide and gold nanourchins has been used here to modify the surface of a screen‐printed carbon electrode to enhance the sensitivity of the electrochemical DNA detection system. A specific single‐stranded DNA probe was designed based on the target DNA sequence and was thiolated to be self‐assembled on the surface of the gold nanourchins placed on the modified electrode. Doxorubicin was used as an electrochemical label to detect the DNA hybridisation using differential pulse voltammetry (DPV). The assembling process was confirmed using scanning electron microscopy (SEM) imaging, cyclic voltammetry (CV), and the EIS method. The high sensitivity of the proposed system led to a low detection limit of 0.16 fM and a wide linear range from 0.5 to 950.0 fM. The specificity of the DNA hybridisation and the signalling molecule (haematoxylin) caused very high selectivity towards the target DNA than other non‐specific sequences.

## 1 INTRODUCTION

DNA sequences are essential biomarkers in medical and biological sciences, and any change in their sequence can be a sign of a biological situation or possibly a disease [1, 2, 3]. However, another application of DNA detection/assessment is to identify or confirm a specific organism in an anonymous sample that can be harmful to health, against a particular country's law or religious beliefs. About the last case, pork meat and other byproducts such as gelatin are banned in religions, and therefore, the detection of those products can be an essential need in their import and marketing strategies [4, 5, 6]. There are methods for assessing or quantifying DNA sequences, including but not limited to electrophoresis, polymerase chain reaction‐restriction fragment length polymorphism (PCR‐RFLP), immunohistochemistry, DNA sequencing, high‐performance liquid chromatographic (HPLC), Liquid chromatography‐mass spectrometry (LC‐MS), biosensors, etc. [7, 8].

DNA biosensors and nanobiosensors combine the advantages such as specificity of DNA hybridisation methods and enhanced sensitivity brought by using nanomaterials. Among different types of nanobiosensors, the electrochemical nanobiosensors of DNA comprise even more advantages, including higher accuracy, lower price, and a more simple fabrication process [9, 10, 11]. That is why electrochemical DNA biosensors have been used mostly in medical and agricultural detection strategies, and their applications are constantly growing day after day [11, 12, 13]. They also proved to be effective in the recent COVID‐19 pandemic for the detection of the virus [14].

So far, a wide range of nanomaterials and composites with different physico‐chemical properties have been used in electrochemical nanobiosensors for surface expansion, accelerating electron transfer, etc. Based on the literature, the most commonly used nanoparticles in electrochemical DNA nanobiosensors are gold nanoparticles and graphene derivatives [10, 15, 16].

Besides the nanomaterials mentioned above, scientists have used gold nanostructures [17]; silver nanoparticles [18]; gold nanosheets [19]; carbon nanotubes [20]; Mesoporous Silica Nanoparticles [21]; DNA nanostructures [22]; nanocomposites [23, 24] and many others for electrochemical nanobiosensors of DNA.

In this research, we have used a combination of graphene oxide and gold nanourchins to modify the working electrode to enhance the sensitivity of the electrochemical nanobiosensor.

The graphene family does have excellent properties that are mostly used among nanomaterials in many fields. They are applied in biosensors with different approaches to enhance the sensitivity, especially in electrochemical biosensors. Many forms of nanomaterials in the graphene family have unique properties that can be used in biosensors for different goals [25, 26, 27, 28]. Electrochemically reduced graphene oxide (ErGO) has a more conductive nature than GO and can be more attractive for nanobiosensors [29]. Gold nanourchins (AuNUs), with their unique shape like nanoantenna‐type spherical particles, have been reported to have specific optical properties [30]. In electrochemistry, the high surface area of these nanoparticles can be an advantage over standard spherical nanoparticles and can be a way to enhance the sensitivity of the nanobiosensor. AuNUs have been used in different electrochemical sensing mechanisms and reported to be effective previously [31, 32, 33]. The ssDNA probes with a thiol functional group at their 5′ end can be self‐assembled onto the gold surface (Self‐assembled monolayers (SAMs) of thiolates on gold surfaces). This phenomenon has been used for the attachment of DNA/RNA onto gold surfaces in many DNA/aptamer biosensors so far based on the formation of a gold–sulphur bond caused by a driving force for the anchoring of thiols on gold surfaces [29, 34, 35]. Doxorubicin is a chemotherapy drug whose intercalating interaction with the DNA is proved in many publications [36, 37, 38, 39]. Also, its electroactive properties can be used for electrochemical measurement of this drug [40] and, more importantly, as an electrochemical label in DNA/RNA biosensors [29, 41].

Although many DNA biosensors have used nanomaterials for signal amplification, we developed an electrochemical DNA nanobiosensor based on novel ErGO and AuNUs to detect pork DNA in food and drug samples in gelatin products. This combination of hybrid nanomaterials, besides the application of doxorubicin as an electrochemical label, is expected to effectively enhance the output signal of the nanobiosensor to detect the lower concentrations of the target DNA. We optimized the fabrication process, checked the specificity and real sample efficiency of the nanobiosensor.

## 2 MATERIALS AND METHODS

### 2.1 Materials and oligonucleotides

At first, we attempted to modify the surface of the screen‐printed electrodes with nanomaterials. Graphene oxide (Catalogue No. 777676) and gold nanourchin, with a diameter of ∼70 nm in 0.1 mM PBS solution with particles 1.2 × 10^16^/ml (Catalogue No. 797731), were both obtained from Sigma‐Aldrich Company, USA. All oligonucleotides used were obtained from Metabion Company, Germany; they were of very high quality and purity and their sequences are reported in Table 1. The bold letters are those that were altered to make a mismatch. Doxorubicin hydrochloride (Dox) was purchased from Ebewe Pharma Company, Austria. All the other materials were purchased from Sigma Aldrich Company, USA.

**TABLE 1:** Sequence of used oligonucleotides for the nanobiosensor fabrication and testing

Oligonucleotide name	Sequence
Target DNA	5′‐TACCATTGAGGGGAGATTTAGGC‐3′
ssProbe	5′‐**SH**‐GCCTAAATCTCCCCTCAATGGTA‐3′
1nt‐mismatch	5′‐TACCATTGAGG**T**GAGATTTAGGC ‐3′
3nt‐mismatch	5′‐TACCAT**C**GAGG**T**GAGAT**G**TAGGC‐3′
Non‐complementary sequence	5′‐GCTACATGGTAACTAGCGATTGA‐3′

### 2.2 Instrument

The Autolab potentiostat/galvanostat PGSTAT101 (Metrohm) was used as the main instrument in which the NOVA 2.1 was the interfacing software. The electrode was screen‐printed carbon electrodes (SPCE, DRP‐C110) from Dropsens, Spain. Scanning electron microscopy (SEM) imaging and energy dispersive spectroscopy (EDS) were performed by Zeiss Sigma 500 VP FESEM device. The Zeiss‐EM10C‐80 KV TEM device completed transmission (TEM) imaging.

### 2.3 Optimization of fabrication

All the elements of the experiment were optimized to obtain a higher output signal, including all the fabrication parameters like concentrations of AuNU, GO, Dox, ssProbe and incubation time of ssProbe, and its hybridisation with target DNA and incubation time of Dox. The optimized values were then used in the fabrication process, explained in the next section.

### 2.4 Fabrication protocol of the sensor

The fabrication processes were started by cleaning the screen‐printed electrodes with double distilled water (DDW). 3.0 μL of 0.9 mg ml^−1^ GO solution was added to the carbon electrode and dried at room temperature, and then washed gently with DDW. Next, 3.0 μL of 100.0 μg ml^−1^ AuNU solution was deposited onto the surface of SPCE/GO and kept isolated to be slowly dried at 25°C and then washed with DDW. The AuNUs are expected to attach to the surface of the GO by hydrogen and electrostatic bonding that are stable enough for the biosensing procedure. After the decoration of the AuNU on the GO layer, the electrochemical reduction process was performed in phosphate buffer pH = 6.5 by 15 cycles of cyclic voltammetry, obtained from a previous publication [29]. When conventional methods characterised the modified electrode, the ssProbe solution (90.0 nM) was poured on the modified electrode and kept in a high humidity container for 75 min. Then, the electrode was immersed in 0.1 mM MCH solution for 5 min and washed again. At this stage, the nanobiosensor is ready to be used to detect DNA.

**FIGURE 1:**
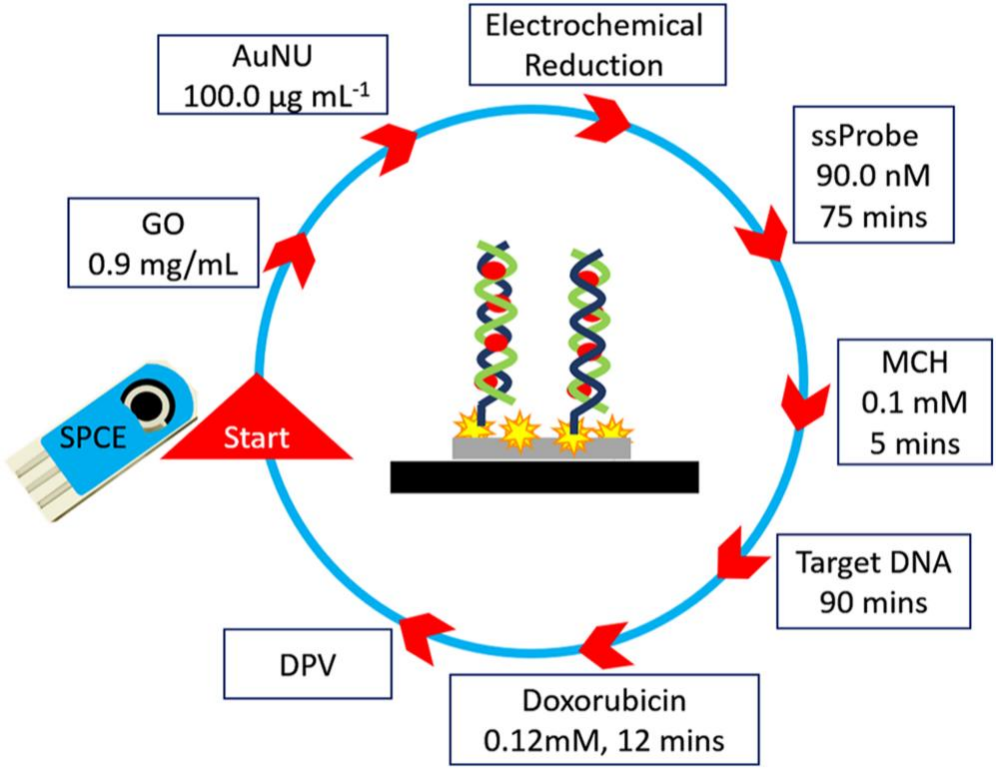
Schematic of sensor fabrication with optimized values of parameters

The hybridisation buffer was then added on the modified electrode of the prepared nanobiosensor and kept isolated for 90 min containing desired concentrations of target DNA. It was then washed, and finally, 0.12 mM Dox solution was placed on the surface of the electrode for 12 min to react with the single or double‐stranded DNA present there. Then, differential pulse voltammetry was performed for the electrode (Potential from +0.650 to +0.265, modulation time = 0.05 s, amplitude = 25 mV, Step Potential = 50 mV, and pH = 7) to measure the peak current of Dox reduction. Cyclic voltammetry and electrical impedance spectroscopy were used to characterise the sensor and FESEM to obtain scanning electron microscopy images of the prepared electrodes with nanomaterials to ensure their presence and coordination. Figure 1 represents the nano‐modified electrode, and its fabrication process is explained here.

### 2.5 Electrochemical characterisation using cyclic voltammetry (CV) and electrochemical impedance spectroscopy (EIS)

To characterise each fabrication step of the nanobiosensor, the electrochemical analyses, including CV and EIS, were performed. Both methods were performed in a solution of 5.0 mM K_3_ [Fe(CN)_6_]/K_4_ [Fe(CN)_6_] containing 0.1 M KCl for each modification of the electrode surface. The CV was performed in a potential range between −0.025 and +0.33 V with a sweep rate of 0.02 V s^−1^.

## 3 RESULTS AND DISCUSSION

### 3.1 Characterisation of fabrication steps

**FIGURE 2:**
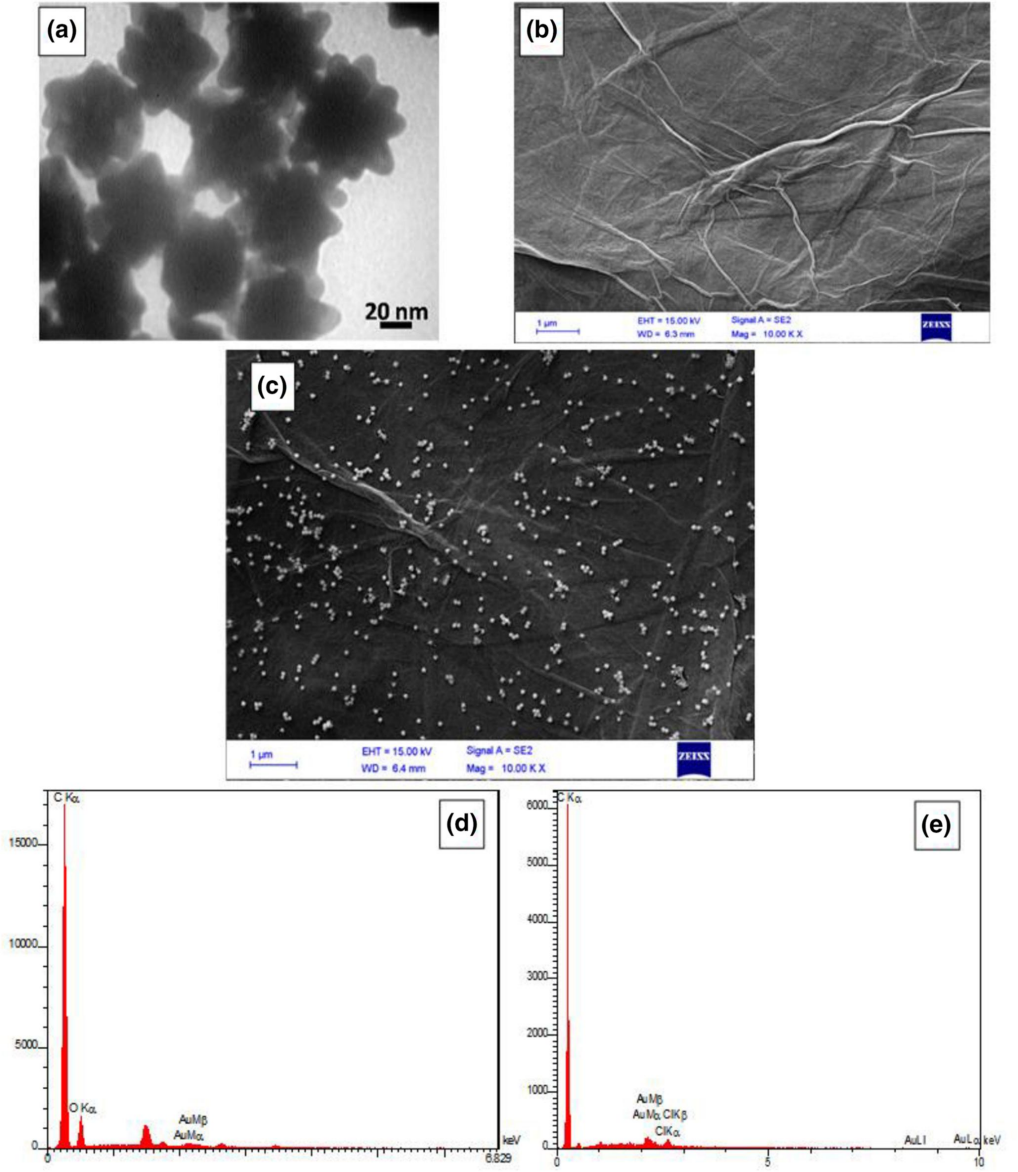
Characterisation of the nanoparticles and electrodes. (a) TEM image of nanourchins. (b) scanning electron microscopyimage of GO surface on the electrode. (c) Nanourchins' dispersion on the GO surface is depicted in the SEM image (d) and (e) EDX analysis of the Go‐AuNU modified electrode before and after electrochemical reduction, respectively

The SEM, TEM and EDS analyses have been carried out to characterise the nanoparticles on the electrodes visually. As shown in Figure 2a, high‐resolution TEM images genuinely confirm the presence of the well‐shaped nanourchins with the correct size as it was purchased. In fact, the urchin‐like shape of the gold nanoparticles is visible in Figure 2a. Also, according to the scale bar of the figure, the average diameter of the nanourchins is around 70 nm, which was mentioned in the product specification.

In addition, the surface of the modified electrode analysed by SEM analysis exhibits both the conformity of the graphene oxide sheet on the SPCE (Figure 2b) and conformal spread of urchins on the sheet after that (Figure 2c). Attachment of the nanomaterials to the surface was to be investigated, and electron microscopy confirmed the attachment and dispersion and maintained the standard shape for both the graphene sheet and the nanourchins. Another defect that can minimise the functionality of the nanomaterials is the agglomeration of the structures, which from the microscopy results, we ensured that no significant agglomeration took place. The distribution of the gold nanourchins on the graphene layer is acceptable, and they formed a dispersed configuration over the graphene to enhance the feature of the nanocomposite.

To assess the presence of nanomaterials on the electrode surface more deeply, the EDX analysis was performed for GO‐AuNU modified electrode before (Figure 2d) and after (Figure 2e) electrochemical reduction process to confirm the efficiency of the electrochemical reduction process. By comparing these two graphs (before and after reduction), it can be seen that the oxygen content decreased significantly (to near zero), which represents a good electrochemical reduction of oxygen‐containing functional groups of GO.

**FIGURE 3:**
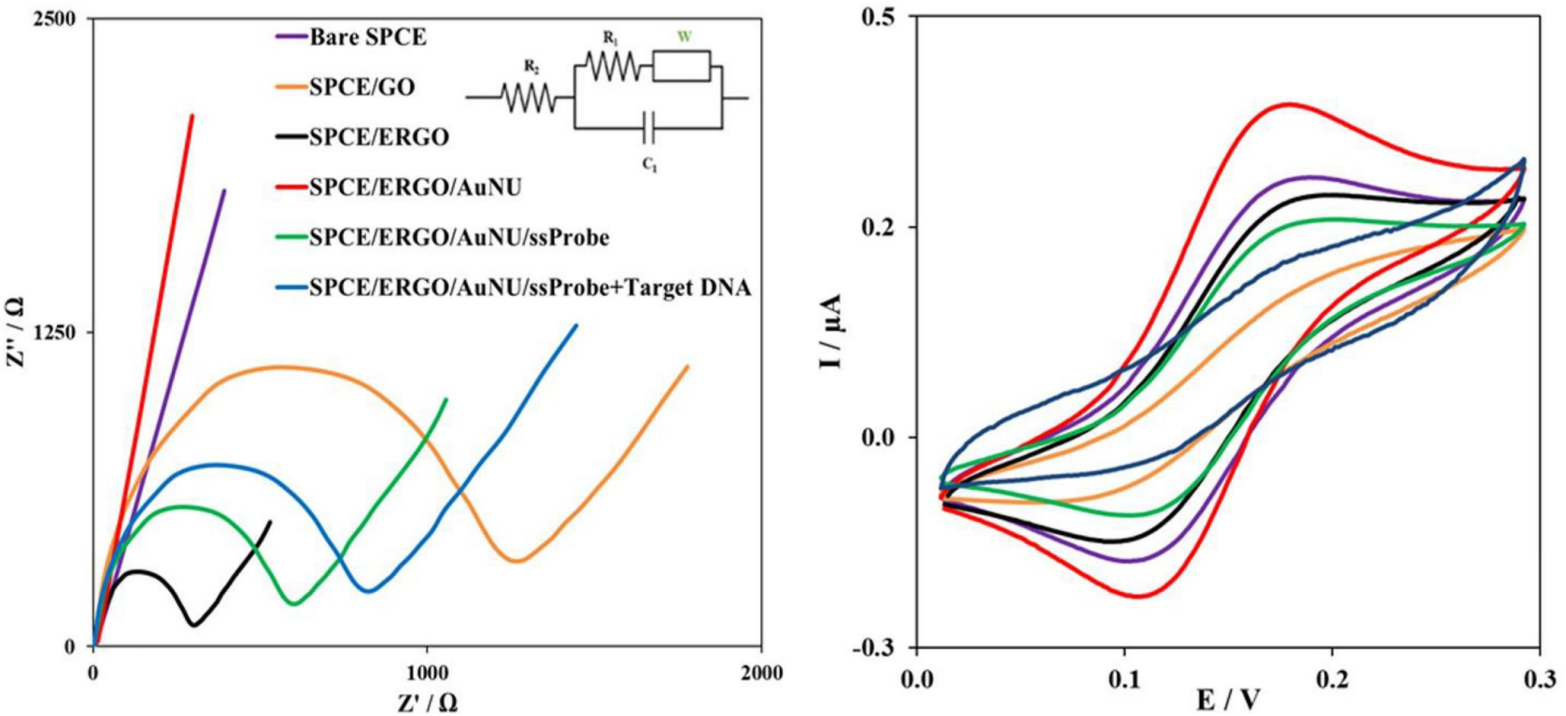
Nyquist plots (left) and Cyclic Voltammetry (right) of the modified electrodes in 5.0 mM solution of K_3_ [Fe(CN)_6_]/K_4_ [Fe(CN)_6_] containing 1.0 M KCl. The curves for each modified electrode are explained in the electrochemical impedance spectroscopy (EIS) figure with a corresponding colour. The equivalent circuit of the EIS study is shown as an inset

For further characterisation of the modified electrode, the cyclic voltammetry and impedance spectroscopy results of the sensor are shown in Figure 3, both performed in the solution of [Fe(CN)_6_]^3−/4−^. The CV results suggested that GO alone significantly decreases the conductivity of the electrode, resulting in the minimum peak current in CV and a high impedance in the Nyquist plot of the GO electrode compared to the bare SPCE electrode. This can be explained because the GO contains functional groups that insulate the electron transfer [42]. However, adding AuNU to the GO also containing an electrochemical reduction, on the contrary, builds up the minimum impedance, and hence the highest peak current in the CV because of the high conductivity of the gold nanoparticles [29]. The electrochemical reduction of the GO, which is decorated by gold nanourchins, significantly increased its conductivity and helped with the peak current in the CV analysis. However, after adding the ssProbe strand, the negative charges of the ssProbe repel the [Fe(CN)_6_] anions, and also, the surface of the electrode is covered, so the electron transfer is more challenging than before. Therefore, the impedance gets higher, and the peak current is decreased in the CV. For the same reason, after adding the target DNA to the electrode surface (higher DNA molecules), the current gets lower and the impedance increases.

### 3.2 Analytical assessment and repeatability of the biosensor

**FIGURE 4:**
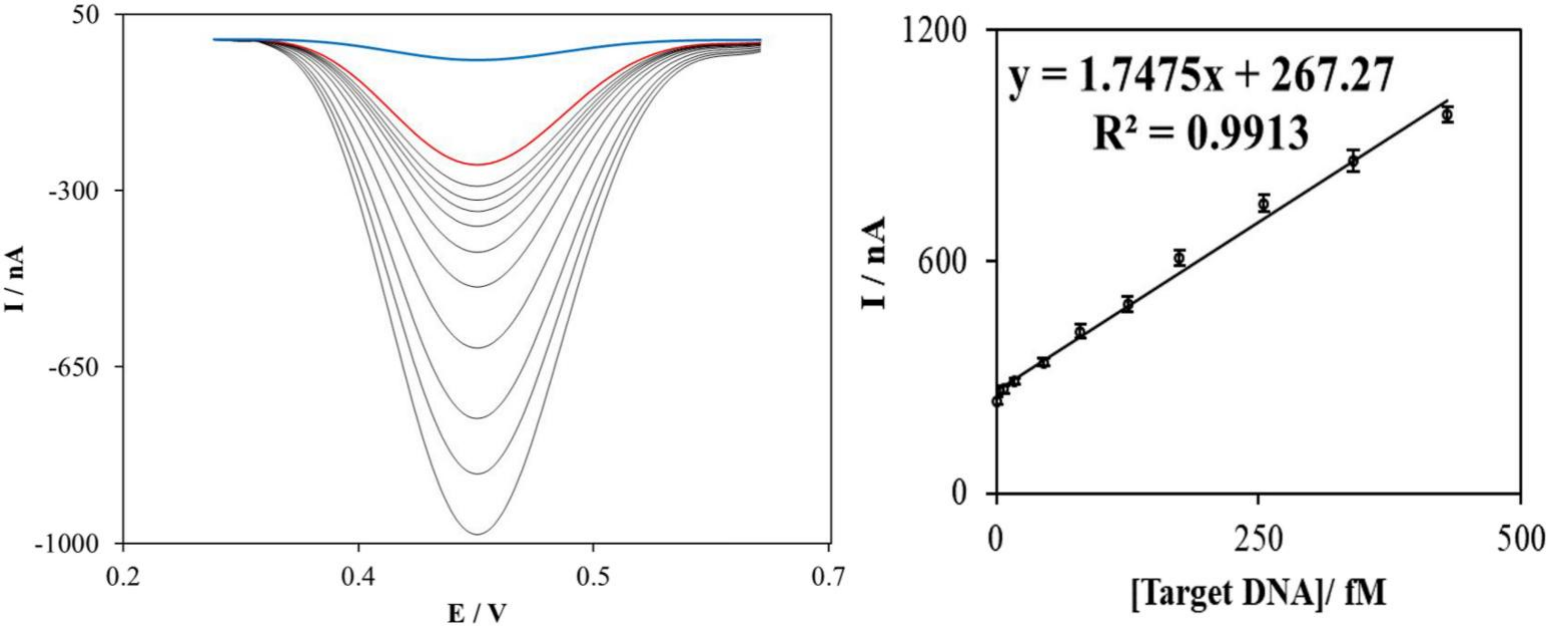
Differential pulse voltammetry analysis of the nanobiosensor for target DNA concentrations, the ssProbe (red curve) and nanomodified electrode (blue curve) (left) and calibration curve (right). DPVs were measured in the phosphate buffer solution pH 7.0 at 25 mV modulation amplitude scanning from 0.65 to 0.25 V, with a step potential of 0.0049

To measure the linear range of the sensor, differential pulse voltammetry has been performed for different concentrations of the target. As shown in Figure 4, the sensor exhibits an exceptionally linear manner in the range of 0.5–950.0 fM of target DNA. The correlation value of the measurements in the linear range is 0.9913, which is near to 1 and shows a linear relationship of the concentrations versus their respective peak currents.

In addition, by using a previously explained detection limit calculation method [43] with S/*N* = 3 and using the slope of the calibration curve, the sensor shows a low detection limit of 0.16 fM of target DNA. Such a wide linear range and low detection limit can signify the sensitivity and functionality of the developed nanobiosensor.

The DPV of the ssProbe‐Dox is also shown in Figure 4 to represent its difference to the ssProbe + target hybrid and is also being used as a negative control. By this comparison, it can be concluded that the intercalation of Dox molecules into double‐stranded DNAs is the reason for this difference in the DPV peak current. This result agrees with the previous publications that explained the intercalation interaction [29, 41]. In addition, the step‐modified process of biosensor fabrication inhibits the Dox molecules to attach to the nanomaterials directly therefore they cannot produce any additional signal. This is because a layer of ssDNA probes was covered on the nanomaterials by the self‐assembling method based on our previous publication [29].

Compared to other biosensors and nanobiosensors that used graphene and gold nanoparticles, we have collected a summary, as shown in Table 2. As it can be seen, the developed nanobiosensor does have advantages over most other similar DNA biosensors. The relativity better LOD might be originating from the shape of the gold nanourchins applied in this research, which might increase the surface area compared to the gold nanoparticles.

**TABLE 2:** Comparing the results of the DNA nanobiosensor to others

Nanomaterials	Limit of detection	Linear range	Amplification	Reference
Graphene	0.15 μM	‐	Loop‐mediated isothermal amplification	[44]
rGO + Au NPs	35.0 aM	0.1 μM–0.1 fM	Auxiliary probe	[45]
Graphene + Gold clusters	0.057 fM	0.02 fM–20.0 pM	Enzymatic	[46]
Graphene + Au nanorod	403 pM	10 pM–10 nM	Auxiliary probes	[47]
rGO + AuNPs	21.3 fM	80.0–1200.0 fM	‐	[15]
Exfoliated GO + AuNUs	13.0 fM	40.0–1100.0 fM	‐	[32]
ErGO + AuNUs	0.16 fM	0.5–950.0 fM	‐	This work

In addition, for all the target DNA concentrations in the linear range, the repeatability of the nanobiosensor for three replications was assessed. In fact, for each concentration, the mean value of the three replications and standard deviation were calculated. The mean value is reported in the calibration curve plot (Figure 4), and their relative standard deviation (RSD) percentage was calculated and reported as error bars in that figure. As it can be seen, the RSD values are low, considering the complicated fabrication process of the biosensors that contain different stages and biological components. The lower RSD value was 4.1% and the highest was 8.3%, which is acceptable.

### 3.3 Selectivity of the biosensor

**FIGURE 5:**
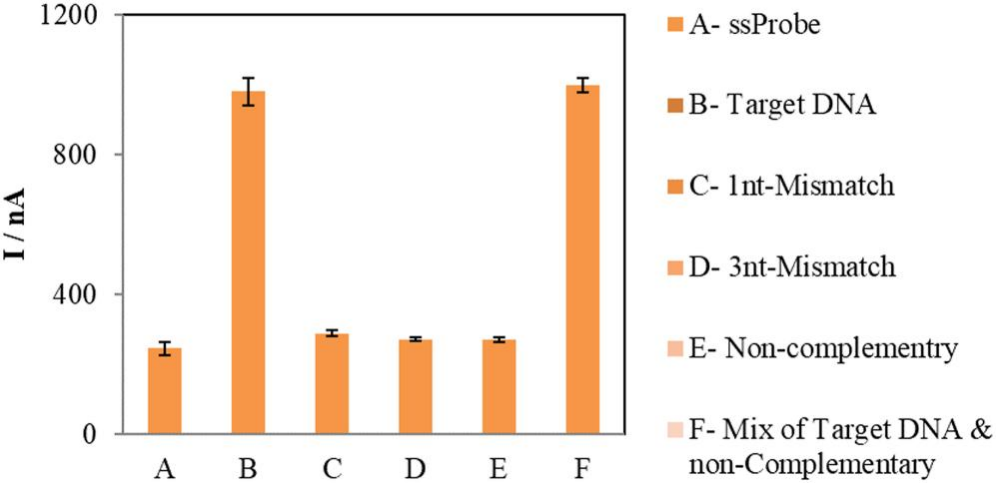
Specificity of the developed nanobiosensor for different DNA samples by showing their differential pulse voltammetry peak currents. DPVs were measured in the phosphate buffer solution pH 7.0 at 25 mV modulation amplitude scanning from 0.65 to 0.25 V, with a step potential of 0.0049

In order to test the specificity of the sensor to the exact target DNA strand, we measured the current response of the sensor comparably for the target DNA and with adjacent DNAs having few mismatches. As depicted in Figure 5, only for the exact target DNA sequence, a sharp current response is observable, and for all the other mismatches, there are no high currents, and all the non‐specifics do not have a significant difference to the sensor response, which is an excellent specificity. Also, we added samples containing both the target and non‐complementary DNA to simulate the real sample environment with many strands present. For this mixed sample, also, due to the presence of the target DNA, a sharp increment in current is seen, ensuring the sensitivity of our tool to the target DNA. The error bars in Figure 5 represent four replications of every reported experiment.

### 3.4 Fabrication process optimization

As it can be seen, the detection mechanism is based on hybridisation of the immobilised ssDNA probe with target DNA and the output signal measured by reduction DPV signal of the Dox and the electrochemical label. The hybrid nanostructure of the ErGO + AuNU was used to enhance the output signal of the nanobiosensor.

**FIGURE 6:**
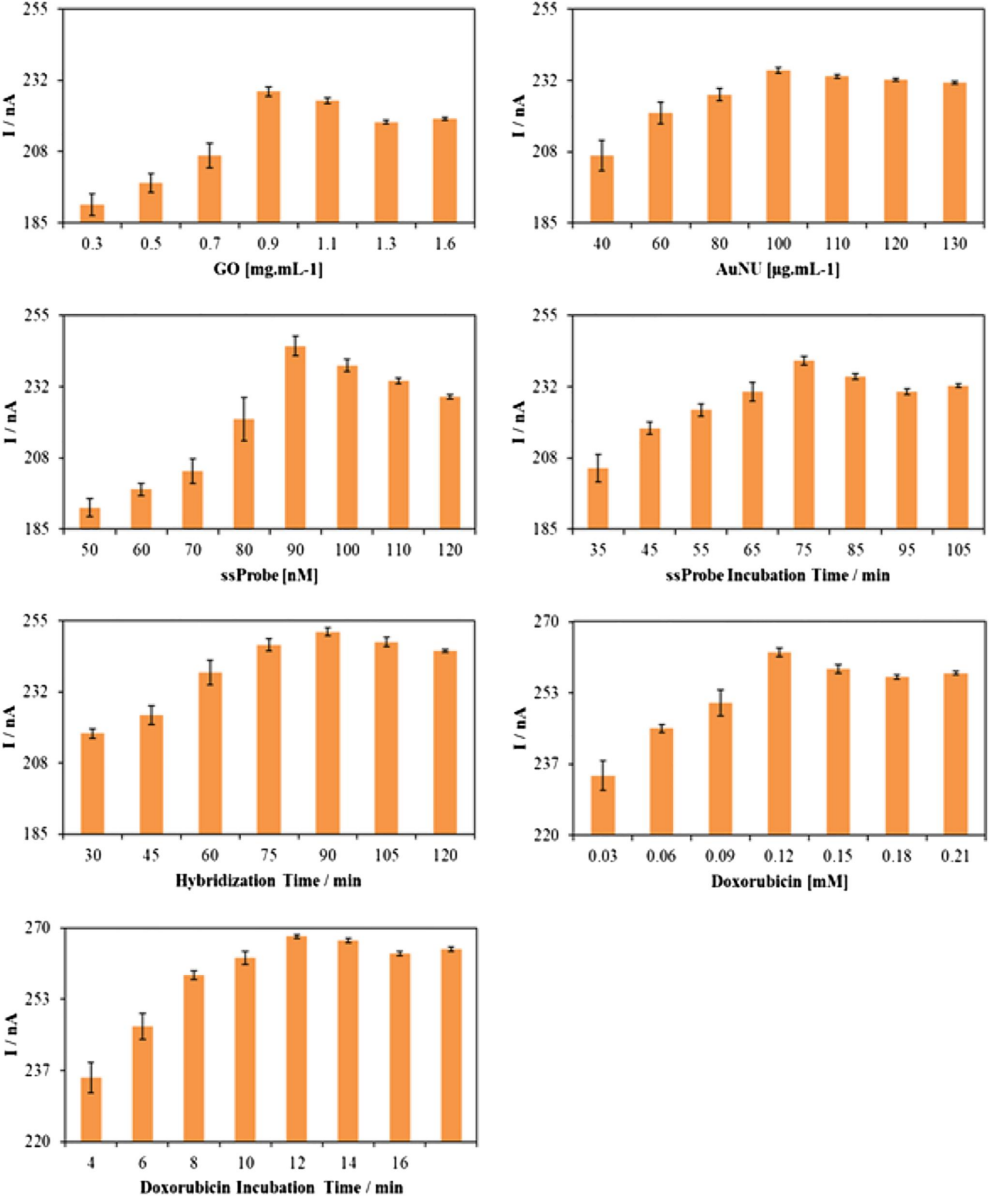
Optimization of important fabrication variables of the developed nanobiosensor

In order to achieve the presented optimized values (Figure 6) of the variables affecting the overall response of the sensor, we measured the current output changing each affecting variable while having all others fixed. The variables to be optimized were in the order of GO, AuNU, and ssProbe concentration, ssProbe incubation and hybridisation time, and at last, the concentration and time for doxorubicin treatment. As seen in Figure 6, a sharp increasing trend to a peak followed by a gradual decrement is observed for all the parameters. For each of them, the peak value has been selected as the optimum value for all the following tests, and the nanobiosensor was fabricated based on those achieved values, as is explained in Figure 6.

### 3.5 Tests in real DNA extraction samples

Finally, to ensure the functionality and test the precision of the sensor, the response for three real samples has been taken. As explained before, we have prepared a sample containing four probable compounds in extracted DNA solutions and added them to our synthetic DNA solution. Then, as shown in Table 3, we did the test for a relatively small (25.0 fM), medium (300.0 fM) and high concentration (900.0 fM) of the target DNA to cover all the actual concentrations ranges. We spiked those DNA concentrations into the prepared samples. As a result, great precision and high recovery percentage with the low error were observed for all three samples.

**TABLE 3:** Real sample spike studies of the fabricated DNA nanobiosensor

Sample	Added DNA (fM)	Detected DNA (fM)	Recovery (%)	Relative standard deviation (%)
1	25.0	25.4	101.6	3.09
2	300.0	296.4	98.8	2.51
3	900.0	902.3	100.2	2.4

## 4 CONCLUSION

We applied a nanocomposite of electrochemically reduced graphene oxide and gold nanourchins on the screen‐printed carbon electrode to detect specific DNA. The combination of these nanomaterials was used to enhance the sensitivity of the detection system. The developed DNA nanobiosensor was very sensitive and selective towards its specific target DNA sequence, and the results of the real sample study were also promising. Using novel nanomaterials, a good intercalating electrochemical label (Dox), screen‐printed electrode, and DPV method could be the ways for superior function and sensitivity over the majority of previous publications. Besides, by considering the low cost, no amplification, and simple fabrication process, it can be concluded that the developed biosensor can be used in food science and technology agents to detect unwanted or dishonest food products, especially gelatin.

## CONFLICT OF INTEREST

The authors declare that they have no known competing financial interests or personal relationships that could have appeared to influence the work reported in this paper.

## Data Availability

Data available on request from the authors.
